# RETRACTION: Effect of Laparoscopic‐Assisted Transvaginal Hysterectomy on Wound Complications in Patients With Early Stage Cervical Cancer: A Meta‐Analysis

**DOI:** 10.1111/iwj.70359

**Published:** 2025-03-18

## Abstract

**RETRACTION**: ZhangJ. , ZhouY. , YeH. , ChenC. , and LuoY. , “Effect of Laparoscopic‐Assisted Transvaginal Hysterectomy on Wound Complications in Patients With Early Stage Cervical Cancer: A Meta‐Analysis,” International Wound Journal 21, no. 4 (2024): e14529, 10.1111/iwj.14529.38069545 PMC10961037

The above article, published online on 08 December 2023, in Wiley Online Library (http://onlinelibrary.wiley.com/), has been retracted by agreement between the journal Editor in Chief, Professor Keith Harding; and John Wiley & Sons Ltd. Following an investigation by the publisher, all parties have concluded that this article was accepted solely on the basis of a compromised peer review process. The editors have therefore decided to retract the article. The authors did not respond to our notice regarding the retraction.

**RETRACTION**: J. Zhang , Y. Zhou , H. Ye , C. Chen , and Y. Luo , “Effect of Laparoscopic‐Assisted Transvaginal Hysterectomy on Wound Complications in Patients With Early Stage Cervical Cancer: A Meta‐Analysis,” International Wound Journal 21, no. 4 (2024): e14529, 10.1111/iwj.14529.38069545 PMC10961037

The above article, published online on 08 December 2023, in Wiley Online Library (http://onlinelibrary.wiley.com/), has been retracted by agreement between the journal Editor in Chief, Professor Keith Harding; and John Wiley & Sons Ltd. Following an investigation by the publisher, all parties have concluded that this article was accepted solely on the basis of a compromised peer review process. The editors have therefore decided to retract the article. The authors did not respond to our notice regarding the retraction.

